# Large language models in the management of chronic ocular diseases: a scoping review

**DOI:** 10.3389/fcell.2025.1608988

**Published:** 2025-06-18

**Authors:** Jiatong Zhang, Xiaoxi Song, Bocheng Tian, Mingke Tian, Zhichang Zhang, Jing Wang, Ting Fan

**Affiliations:** ^1^ The First Clinical Medical School, China Medical University, Shenyang, China; ^2^ Liaoning Education Informatization Development Center, Liaoning Institute of Education, Shenyang, China; ^3^ The Second Clinical Medical School, China Medical University, Shenyang, China; ^4^ Emory College of Arts and Sciences, Emory University, Atlanta, United States; ^5^ School of Intelligent Medicine, China Medical University, Shenyang, China; ^6^ The Department of Ophthalmology, Shengjing Hospital of China Medical University, Shenyang, China

**Keywords:** large language models, chronic ocular diseases, multimodal data, clinical decision support, full process management

## Abstract

Large language models, a cutting-edge technology in artificial intelligence, are reshaping the new paradigm of chronic ocular diseases management. In this study, we comprehensively examined the current status and trends in the application of large language models in major blinding chronic ocular diseases such as glaucoma, cataract, and diabetic retinopathy through a systematic scoping review approach. We conducted this review based on the Preferred Reporting Items for Systematic Reviews and Meta-Analyses extended to characterize the application of large language models in the field of chronic ocular diseases. The study reveals that large language models demonstrate comparable efficacy to experts in disease screening, diagnostic decision-making, personalized precision treatment recommendation, and accessibility of healthcare resources by integrating multimodal clinical data. However, the application of the technology still faces a triple challenge: (1) the limitation of model generalization ability due to the multimodal nature of clinical data; (2) the ethical controversy caused by the insufficient interpretability of algorithms; and (3) the lack of a standardized validation framework. Future directions emphasize the need for specialized model training, multimodal algorithm optimization, the establishment of a multinational multicenter clinical validation platform, and the construction of an ethical framework for dynamic regulation. Large language models are expected to evolve from an assisted decision-making tool to a core component of precision medicine for chronic ocular diseases, and ultimately to achieve an ecosystem of energy-efficient full-cycle management of chronic ocular diseases.

## 1 Introduction

### 1.1 Background

There has been a global surge in chronic ocular diseases, and the two iconic diseases, cataract and glaucoma, are the two leading causes of blindness globally (Huang Y. J. et al., 2024). Some studies show that global cataract patients reached about 94 million in 2020, and glaucoma patients rose from about 76 million in 2020 to about 111.8 million in 2024 (Hu and Wang, 2022; Su et al., 2025). And chronic ocular diseases have atypical symptoms in the early stage and diverse symptoms in the progressive stage (Delsoz et al., 2023), characterized by high blindness, long duration of disease, and urgent need for patient education, and uneven distribution of ophthalmic specialty medical resources globally, the traditional management model is faced with the challenges of low follow-up adherence and limited access to health information (Goktas, 2025).

With the rapid development of artificial intelligence technology, large language models (LLMs), such as ChatGPT-4 and PaLM, are reshaping the service model in the healthcare field by virtue of their powerful natural language processing and generative capabilities (Shi et al., 2024). In the field of chronic ocular diseases management, which requires long-term follow-up and personalized interventions, LLMs show remarkable potential. LLMs are expected to provide innovative solutions for early intervention of chronic ocular diseases, patient self-management, and telemedicine through intelligent questioning, health counseling, and medical record analysis (Wang et al., 2025).

In recent years, studies have been conducted to explore the application of LLMs in the areas of ophthalmic image recognition and risk prediction, but the comprehensive value of LLMs in the chronic ocular diseases full process management (e.g., patient-doctor communication, medication guidance, and behavioral interventions) has not yet been evaluated in a systematic manner (Li J. J. et al., 2024; Shaheen et al., 2025). Most of the existing reviews focus on the technical aspects or acute ocular diseases, and lack a comprehensive overview of the application scenarios, practical effects, and ethical risks of LLMs in chronic ocular diseases. In addition, the differences in the applicability of LLMs in diverse populations (e.g., the elderly, patients with low health literacy) and their integration paths with existing healthcare systems still need to be further explored (Zhang et al., 2025).

### 1.2 Aims

**FIGURE 1:**
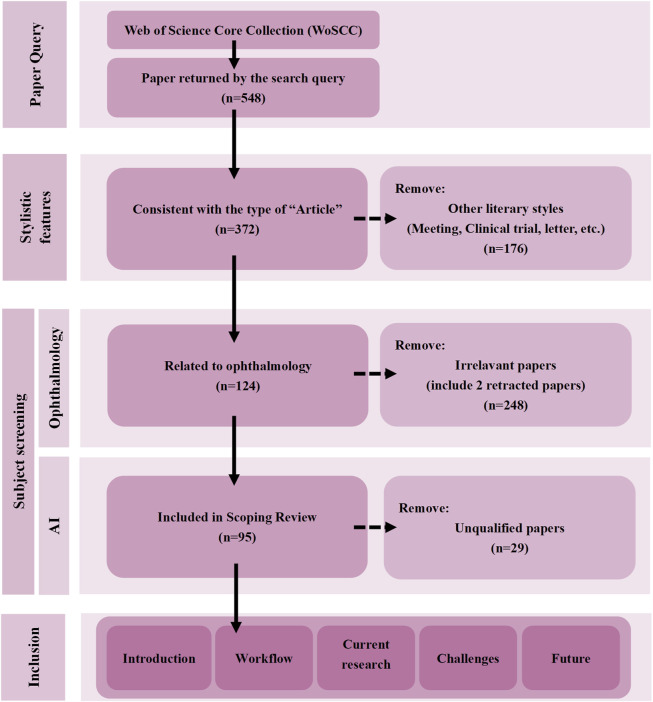
Overview of the scoping review of PRISMA extension for scoping reviews (PRISMA-ScR) process.

The aim of this study is to comprehensively assess the current status and development trend of the application of LLMs in the management of chronic ocular diseases through a systematic scoping review approach in the Preferred Reporting Items for Systematic Reviews and Meta-Analyses (PRISMA) (Figure 1). Specific objectives include (1) to sort out the key technical pathways and application scenarios of LLMs in the management of major chronic ocular diseases (e.g., glaucoma, cataract, diabetic retinopathy, and telltale myopia, etc.); (2) to analyze the major technical bottlenecks and barriers to clinical translation in the current applications; and (3) to explore the future directions of development, including optimization of the specialization model, the establishment of a multicenter validation framework, and the development of ethical norms (4) to discuss the future development direction, including the optimization of specialized models, the establishment of multi-center validation framework, and the formulation of ethical norms. Through this review, we expect to provide theoretical references for subsequent studies and promote the standardized application and innovative development of LLMs in chronic ocular diseases management.

This study utilized a scoping review approach rather than a traditional systematic evaluation based on the following considerations: first, the application of LLMs in chronic ocular diseases is an emerging field and the research evidence is still in a rapid developmental stage; and second, the methodology allowed us to capture the diversity of technological development in a more holistic manner and not only confined to the assessment of efficacy. Through this approach, we are able to better grasp the full picture of research and trends in this cross-cutting area.

## 2 Workflow of LLMs in chronic ocular diseases

**FIGURE 2:**
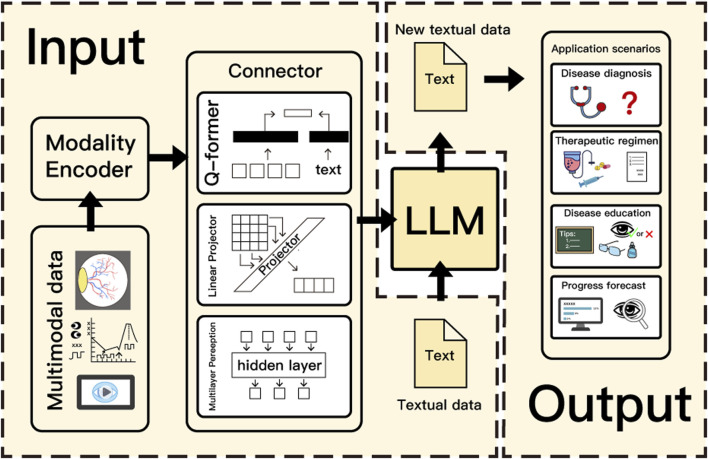
Operating principle of LLMs in chronic ocular diseases.

Nowadays, the integration of multimodal algorithms with LLMs opens up new opportunities for healthcare with chronic ocular diseases. As an input part, textual data can be directly input into the large model, while data in other modalities, such as fundus photography, electro-oculogram, and related videos, need to be imported into the modality encoder for processing, and then imported into the connector to be converted into a form that can be recognized by the LLMs. Q-former, linear projector, and multilayer perception (MLP) are three common types of connectors. Q-former is a widely used mapping network that compresses redundant information through feature alignment. Linear Projector uses matrix operations to project other modal data into the same dimensions as the LLMs (Li Y. X. et al., 2024). multilayer perception is better at processing nonlinear features, such as image segmentation (Gao et al., 2023). LLMs output results based on the input data, and are applied to different scenarios in chronic ocular diseases such as disease diagnosis, therapeutic regimen, disease education, and disease progression forecast (Figure 2).

**FIGURE 3:**
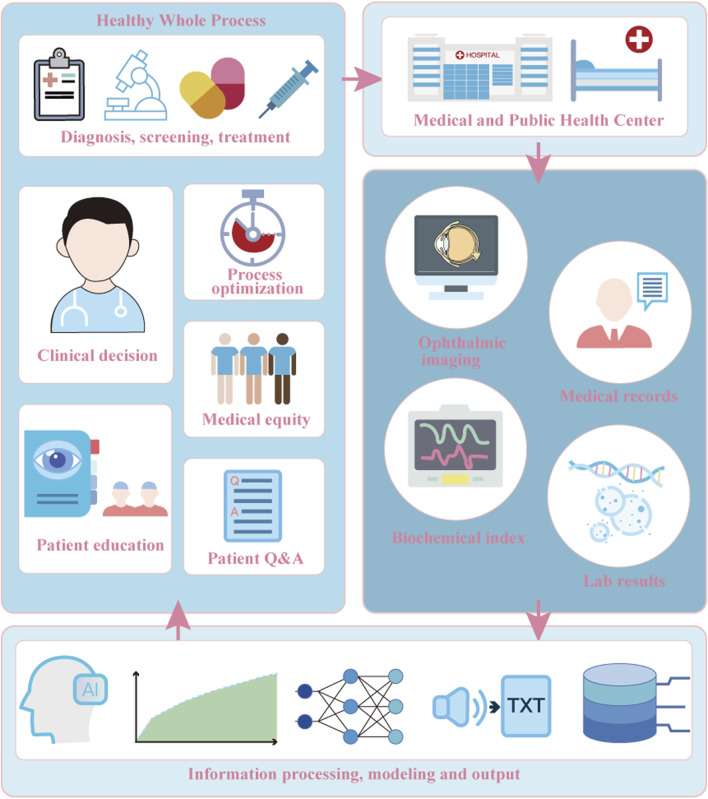
Workflow of LLMs in chronic ocular diseases.

The training process of medical LLMs is a continuous iterative process between the medical data side and the computer side. A certain amount of data is the basis for the training of the LLMs. Figure 3 describes the workflow of medical LLMs in chronic ocular diseases. The researcher obtains electronic health records (HERs) that have been privacy desensitization protected from relevant databases or healthcare centers to extract structured data, such as demographic information, examination results, and unstructured data, such as free text medical records, fundus images, surgical reports, and other multimodal data, and filters and cleanses them in accordance with certain criteria, to create an initial database for training (Son et al., 2021; Hu and Wang, 2022; Yu et al., 2022; Maywood et al., 2024; Kang et al., 2025).

The data will be imported into the computer side, and techniques such as self-attention mechanism and feed-forward neural network will be utilized to architect general-purpose LLMs (e.g., BERT, ChatGPT) or pre-training models in the medical field, and there are also some researches that incorporate computer vision models to achieve multimodal recognition (Hu and Wang, 2022; Yu et al., 2022; Mihalache et al., 2024; Sensoy and Citirik, 2024). According to the application scenarios of macromodels (e.g., surgery prediction, patient questions and answers (Q&A), disease progression inference, etc.) the database is utilized to form macrolanguage models adapted to the specific domains by performing fine-tuning, reinforcement learning and other steps (Hu and Wang, 2022; Yu et al., 2022; Spina et al., 2025). Combine the trained bigram models against human experts to confirm the model performance (Delsoz et al., 2024; Huang A. S. et al., 2024), then put into clinical guidance or patient education to generate diagnosis and treatment recommendations. It is applied to scenarios such as assisting in the diagnosis of glaucoma (Carlà et al., 2024) and generating educational materials for chronic ocular diseases (Spina et al., 2025), while users such as healthcare organizations collect patient feedback to form a new database, and reintroduce the database into the LLMs for continuous iterative training to optimize performance.

## 3 The current research of LLMs in chronic ocular diseases

The scoping review based on a systematic review of the global evidence that the use of LLMs in chronic ocular diseases management is characterized by a dichotomy of “technological acceleration” and “clinical lag”. Supplementary Table 1 shows the majority of existing LLMs in chronic ocular diseases management, and the results are used to deconstruct the current status of LLMs in screening and diagnosis, clinical support, and health equity. Table 1 lists the performance metrics and explanations for evaluating large models that appear to be commonly used in the field of chronic ocular disease.

**TABLE 1:** Metrics for evaluating LLMs performance and their definitions.

Metric	Definition
AUROC	Area Under the Receiver Operating Characteristic Curve, measuring overall classification performance
BLEU	Bilingual Evaluation Understudy, evaluating machine translation by comparing the n-gram overlap between the generated text and the reference text. It calculates the accuracy of different N-grams and introduces short sentences. The range is usually between 0 and 1, with higher values indicating higher similarity
C_1_	Reflects Clinical and Scientific Consensus, whether the response aligns with medical and scientific consensus
C_2_	Likelihood of Possible Harm, risk of the answer causing harm to patients
C_3_	Evidence of Correct Reasoning, logical rationale behind the answer
C_4_	Evidence of Correct Comprehension, accurate understanding of the question
C_5_	Evidence of Correct Retrieval, use of relevant and accurate medical knowledge
C_6_	Missing Important Content, omission of critical information
CIDE	Consensus-based Image Description Evaluation, mainly used for image description tasks. It generates similarity between text and multiple reference texts by weighted calculation, emphasizing semantic diversity and consensus. There is no fixed range for CIDE scores, but they are usually between 0 and 10, with higher scores indicating better semantic matching
Cohen’s Kappa	A statistical measure that quantifies inter-rater reliability (agreement between evaluators) while accounting for chance agreement
CRIE	Chinese Readability Index Explorer, a computational tool designed to assess the readability of Chinese texts. It integrates 82 multilevel linguistic features (e.g., word frequency, sentence complexity, semantic cohesion) to generate readability scores. These scores categorize texts into grade levels: Levels 1–6: Elementary school, Levels 7–9: Middle school, Levels 10–12: High school
DISCERN	DISCERN Quality Criteria for Judging Patient Information About Treatment Choices, a validated tool for evaluating the quality of health information, particularly patient education materials
EQIP	Ensuring Quality Information for Patients, evaluating the quality of written medical information to ensure that the content is patient friendly, accurate and easy to understand. Contains 20 questions, with a “yes/no” answer score, with a maximum score of 100
F1 Score	Harmonic mean of precision and recall, suitable for imbalanced datasets
FK	Flesch-Kincaid, a tool that quantifies the difficulty of reading text to assess the readability of responses from different sources on a scale of (0-100), with higher scores indicating easier reading of the text
FKGL	Flesch Kincaid Grade Level, measuring text readability difficulty, aligning with U.S. grade levels.Formula based on average sentence length and syllables per word,0–18, higher scores indicate greater difficulty
FRE	Flesch Reading Ease, assessing the readability of a text, indicating how easy or difficult it is for readers to understand the content, 0–100 (higher scores = easier to read)
GQS	Global Quality Score, a 1–5 point scoring system designed to evaluate the overall quality of clinical recommendations generated by large language models
Lenient F1	Allow partial overlap or approximate matching (e.g., partial coverage of a concept is considered correct). Focus on detecting the presence of concepts, relaxing positional precision
Likert scale	A psychometric tool designed to measure subjective attitudes or opinions using a graded response system (e.g., 1 = “Strongly Disagree” to 5 = “Strongly Agree”). It quantifies responses through averaged scores or frequency distributions, widely applied in surveys to assess patient satisfaction, educational outcomes, or accuracy of information in clinical studies
PEMAT	Patient Education Materials Assessment Tool, a systematic method to evaluate and compare the understandability and actionability of patient education materials. A higher score means that it's easier to understand and easier to act on
PEMAT-A	Patient Education Materials Assessment Tool for Actionability, evaluating whether the material is effective in guiding the patient to specific actions (e.g., treatment steps, lifestyle adjustments). Evaluation content (7 criteria): clear action suggestions, concrete step breakdown, resource support
PEMAT-U	Patient Education Materials Assessment Tool for Understandability, assessing whether medical education materials are easy to understand by patients, focusing on the language, structure and information presentation of the materials. Evaluation content (17 criteria): language simplicity, structure clarity, focus, AIDS: use diagrams, examples, etc., to help understanding
SMOG	Simple Measure of Gobbledygook, an index used to assess the readability of a chapter or text. It is calculated on the basis of sentence length and complexity in a text. The reading difficulty of an article is estimated by counting the number of multi-syllabic words in the article. The higher the SMOG index, the more difficult it is to read the article. Generally speaking, articles with SMOG scores between 7 and 12 are considered easy to understand, while articles with scores above 12 are more difficult to understand
SOLO	Structure of Observed Learning Outcomes, an educational assessment framework designed to describe the cognitive complexity of learning outcomes. The SOLO taxonomy uses a 1 to 5 scoring scale, corresponding to its five hierarchical levels of cognitive complexity:1 (Prestructural): Responses are irrelevant or show no meaningful understanding, 2 (Unistructural): Addresses a single relevant point but lacks depth or coherence, 3 (Multistructural): Includes multiple relevant points without effective integration, 4 (Relational): Connects ideas logically into a cohesive explanation, 5 (Extended Abstract): Extends understanding to abstract generalizations or novel insights
SPICE	Semantic Propositional Image Caption Evaluation, which evaluates the semantic accuracy of generated text by constructing scene diagrams, including the matching of objects, attributes, and relationships. SPICE also ranges from 0 to 1, with higher values indicating more semantic accuracy
Strict F1	Requires exact boundary matching between predicted concepts and gold-standard annotations (start and end positions must be identical). Evaluate precise localization of concept boundaries

### 3.1 Diagnostics and screening

The technological evolution of LLMs in glaucoma diagnosis and treatment presents a clear innovation path. From unstructured text parsing to multimodal fusion, from single prediction task to complex clinical decision support, an intelligent diagnosis and treatment ecosystem is gradually constructed. Early on, the potential of LLMs to integrate fragmented clinical information, such as the prediction of surgical demand based on the BERT model (AUC = 73.4%), was verified by mining free text in electronic health records, laying a methodological foundation for subsequent technology applications (Hu and Wang, 2022). Then, the technology rapidly penetrates into the core diagnostic process, and several studies have shown that LLMs reach the level of residents in terms of triage accuracy (77.9%) and cases (72.7%), initially realizing the transformation of the role from data tool to residents (Delsoz et al., 2023; Ming et al., 2024). In 2024, multimodal technology breakthroughs become a key turning point, with multi-modal large language models (MLLMs) combining retinal images with clinical texts to significantly improve their analytical capabilities and demonstrate a high degree of synergy with expert decision-making in complex scenarios such as surgical planning (58% match rate), driving the technology to make the leap from assistive to collaborative (Carlà et al., 2024; Ghalibafan et al., 2024). Currently, the application of the technology is further focusing on clinical operability, with innovative solutions such as the code-free risk assessment tool, which transforms the complex algorithms into an intuitive clinical decision support system (Choi and Yoo, 2025). This process not only reflects the technological upgrading of LLMs from an edge tool to the core of diagnosis and treatment, but also redefines the standard of precision in glaucoma diagnosis and treatment through the ability of dynamic learning and integration of multi-source data, and opens up a new paradigm of personalized and efficient ophthalmic medicine.

Diabetic retinopathy (DR) is also one of the leading causes of blindness globally (Wan et al., 2021), and the development of LLMs technology in DR diagnosis and treatment demonstrates a technological evolution from data parsing to multimodal synergy. In the early stage, LLMs took the lead in solving the problem of standardization of DR clinical data, Yu et al. used the BERT model to accurately extract DR lesion features from unstructured fundus reports with a conceptual extraction F1-score of 0.9645, laying the foundation for subsequent automated diagnosis (Yu et al., 2022). On this basis, a convolutional neural networks model combining DR classification and lesion segmentation verifies the potential of multi-task learning to improve the efficiency of DR analysis (Hemelings et al., 2021). With the technological advancement, generative LLMs further empower the full process management of DR, and the ChatGPT model is not only close to the expert level of accuracy in identifying symptoms and treatment options (score 4.84/5), but also can be used for DR severity by automatically annotating DR severity in unstructured medical records (Cohen’s kappa 0.975) (Jaskari et al., 2024; Subramanian et al., 2024). Ultimately, the generative MLLMs, constructed by integrating fundus images with verbal interactions, improved DR screening accuracy from 81% to 92.3% for junior doctors and improved patient by dynamically generating personalized recommendations adherence (p < 0.05) (Li J. J. et al., 2024), highlighting its complete technological closure from data-driven to clinical landing.

LLMs technology also shows potential in the clinical diagnosis and screening of other chronic ocular diseases, such as age-related macular degeneration (AMD) and cataracts. Deep learning models can automatically detect cataract subtypes through images, and the performance is comparable to that of human experts, effectively assisting in early screening (Rampat et al., 2024). In the diagnosis of AMD, the feature fusion framework combines convolutional neural networks to achieve five-level classification of macular lesions, and significantly improves the detection rate of early lesions (Sun et al., 2023). Although these technologies still need to address challenges, their potential in improving screening efficiency, reducing the missed diagnosis rate, and assisting in the analysis of complex cases has been preliminarily verified.

### 3.2 Clinical decision support and process optimization

LLMs technology systematically optimizes ophthalmic clinical practice through three major pathways: assisted treatment planning, automated medical record generation, and multimodal data integration. First, in assisting treatment decision-making, LLMs predicts the need for glaucoma surgery by parsing unstructured clinical texts, simulates clinical thinking, and further integrates fundus images and electronic health records (Hu and Wang, 2022; Delsoz et al., 2023). Notably, ChatGPT-4o has demonstrated high accuracy in pediatric myopia management through structured analysis of disease etiology and symptoms, while maintaining guidance recommendation safety under professional supervision (Kang et al., 2025). In the field of automated medical record generation, LLMs generate discharge summaries and procedure codes with 88% accuracy (Lee et al., 2023; Singh et al., 2023), significantly reducing clinical paperwork burden. Ultimately, through multimodal synergy (e.g., GPT-4V combined with fundus images to achieve international classification of diseases coding) LLMs build a full-cycle support system from screening to closed-loop management (Ghalibafan et al., 2024). LLMs not only optimize the efficiency of individual diagnosis and treatment, but also promote the comprehensive transformation of ophthalmic diagnosis and treatment to precision, efficiency and systematization.

### 3.3 Patient education and health equity

The evolutionary lineage of LLMs technology in ophthalmic patient education clearly demonstrates the technological leap from basic functionality to deep integration. Applications focused on generating personalized health materials, such as using ChatGPT-4 to simplify glaucoma literature to a fifth-grade reading level, significantly improving readability while ensuring content rigor (Spina et al., 2025) In answering frequently asked questions, LLMs have evolved from the initial accurate Q&A to clinical decision support tools, such as integrating multi-source data to construct a glaucoma risk scoring system (Cheong et al., 2024; Choi and Yoo, 2025). Breakthroughs in multi-language support further promote health equity, such as the DeepDR-LLM, which provide low-cost in resource inequality areas through language adaptation and localized output, high-precision medical information support for resource-unequalized areas, and reconstruct the accessibility framework of global health education (Huang A. S. et al., 2024).

## 4 The challenges of LLMs in chronic ocular diseases

**FIGURE 4:**
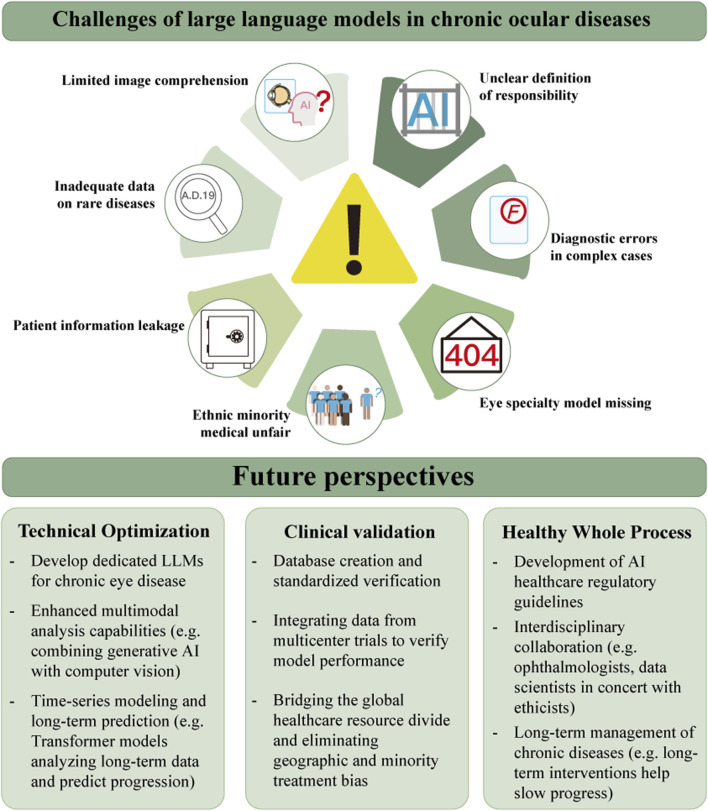
Challenges and future of LLMs in chronic ocular diseases.

Currently there are many applications of LLMs in the field of chronic ocular diseases, while there are still many challenges. Figure 4 exhibits the limitations of today’s LLMs applications in chronic ocular diseases scenarios, which are mainly the technical limitations such as image interpretation capability, data obsolescence, privacy risk, and algorithmic fairness, and the incompleteness of legal, ethical, and fairness such as attribution of responsibility, artificial intelligence (AI) hallucinations, and low coverage of the diseases.

### 4.1 Technical limitations

Clinical data tend to be multimodal and nonstandard, especially ophthalmic free text features tend to be characterized by a lack of attention to grammar, rich in long strings of terms and phrases, and low logic between sentences. Clinical data, such as symptoms, examination results, etc., suffer from the problem of being difficult to embed into models (Hu and Wang, 2022). MLLMs drastically improve the problem, but the development of more adaptive models helps to transform ophthalmic multimodal data into high-quality recognizable feature data, thus circumventing the manual annotation that requires senior ophthalmologists and often inaccurate annotation (Zhao Z. W. et al., 2024). At the same time, the characteristics of ophthalmic clinical data such as difficult access and long time span will make it possible for LLMs to have multi-step inference difficulties in training (Ghalibafan et al., 2024). Currently, some studies have reported attempts to extract information from generalized data based on labeled or unlabeled data with noise (Son et al., 2021). LLMs utilizing unlabeled data for image analysis may be a future direction (Zhao Z. W. et al., 2024). It has been suggested that the development of deep image learning algorithms may change the landscape of management of various ocular diseases (Ghalibafan et al., 2024).

LLMs have the same limitations in terms of accuracy and reliability. LLMs have lower accuracy and answer comprehensiveness for open-ended questions, especially complex questions, and are not yet able to meet clinical requirements (Balci et al., 2024). Fortunately, ChatGPT, Google Bard have shown high performance in the field of zero-shot learning (Cheong et al., 2024). ChatGPT has performed close to the clinician level in some aspects of the Q&A session, far beyond other LLMs models (Delsoz et al., 2023; Ghalibafan et al., 2024). A common problem is the possibility of hallucinations, such as fabricating literature to justify one’s generated text, and clinicians consume a a lot of work to recognize AI hallucinations (Delsoz et al., 2023; Wu et al., 2024). Also models such as ChatGPT may give unavailable treatment options without emerging viable treatment options (Maywood et al., 2024). This uncertainty and lack of global interpretability limits the use of LLMs in clinical practice.

Surprising progress has been made in generating text for ChatGPT responses, with some studies noting that low text readability has been reduced to a fifth grade level, but there are still some scenarios where the readability of the text has limitations and requires a high level of knowledge background, potentially hindering the use of LLMs at the patient education level (Spina et al., 2025). In recent years, the performance of LLMs has gradually improved, providing the ability to provide relatively reliable diagnostic support in many chronic ocular diseases scenarios, but the high standards of medical scenarios make it impossible for LLMs to take the place of physicians yet (Ghalibafan et al., 2024). At present, the application of LLMs to chronic ocular diseases is still in its infancy, and there have been attempts to combine time-series data analysis in cardiology and other fields, but there have not been many reports on the research of combining time-series data analysis in the field of ocular diseases (Ding et al., 2024). The analysis of time-series data in EHR by LLMs. The improvement of the ability of LLMs to analyze time-series data in EHR may bring a new revolution of AI diagnosis and treatment.

### 4.2 Imperfect regulations and ethics

LLMs have become a powerful tool for healthcare and need to be robustly evaluated for functionality and reliability (Bahir et al., 2025). However, the industry lacks uniform norms to test the accuracy of the text generated by LLMS, while also holding them accountable for the recommendations it provide (Yilmaz and Dogan, 2025). For numerous uncommon diseases, the lack of training data for the models leads to poor performance, and the selection of training samples may cause bias in conclusions about minorities (Chang et al., 2024; Ghalibafan et al., 2024). Regional epidemiologic variability was also rarely considered in the studies included in the review. Such differentiation may influence modeling judgments.

In addition, medical data are strictly regulated and their accessibility may receive limitations. LLMs training involves a large amount of clinical information, and the training process often adopts patient data anonymization and data desensitization and encryption to isolate patient privacy and the clinical data itself, which can also safeguard the privacy and security of patients in the later application of LLMs (Ghalibafan et al., 2024). However, due to the complexity of the internal algorithms, researchers still have to be aware of the ethical risks of data leakage and privacy breaches of individual processes. Half of the articles in our scoping review expressed “caution” about the application of LLMs in medical practice, and it is clear that although there are breakthroughs in LLMs in the field of chronic ocular diseases, LLMs models may require higher capabilities to perform specific clinical tasks due to the specificity of clinical medicine.

## 5 Future perspectives

In recent years, LLMs have been developing and gradually applied in chronic ocular diseases. With the optimization of related technologies, the completion of clinical feasibility validation, and the improvement of policy system support in the future (Figure 4), LLMs will build a new ecology for the diagnosis and treatment of chronic ocular diseases.

Although LLMs have achieved the initial application of multimodal integration and dynamic learning in chronic ocular diseases management (Zhao Z. W. et al., 2024; Choi and Yoo, 2025), there is still a need to break through the problems of insufficient depth of ophthalmic specialties (Li J. J. et al., 2024), cross weak generalization of modal alignment algorithms and lack of systematic support for dynamic learning mechanisms (Alqudah et al., 2024). In the future, we need to develop more ophthalmology-specific LLMs to explore the depth of knowledge through specialized model training; develop ophthalmology-oriented multimodal alignment architectures, such as combining generative AI and computer vision to strengthen the multimodal analysis capability; use time-series modeling techniques such as the transformer timing model to predict the long-term disease progression, while designing a closed-loop dynamic learning system to integrate incremental learning and real-time data streams, breaking through the limitations of knowledge curing in static models, overcoming terminology and contextual complexity, and upgrading LLMs from an assistive tool to the core of domain-wide decision-making, and ultimately realizing high-precision, low-cost chronic ocular diseases prevention networks to bridge the global challenge of uneven distribution of healthcare resources.

LLMs have achieved milestones in the auxiliary diagnosis of chronic ocular diseases (Delsoz et al., 2024), but their clinical application still faces two key challenges: the lack of data on rare diseases and standardized validation. In the future, we need to design prospective clinical trials, validate the effectiveness of LLMs in the real world, and solve the problem of insufficient data on rare diseases through synthetic data, while relying on the international collaborative network to achieve multicenter collaboration (Gong et al., 2024), and unify the labeling specifications to establish a high-quality database of chronic ocular diseases in multiple regions and all types of diseases, in order to eliminate the geographical and minority diagnostic and treatment bias. geographic and minority diagnostic bias. Such systematic validation will facilitate LLMs to bridge the global healthcare resource divide and achieve innovation in the whole chain of chronic ocular diseases from screening to personalized management.

Although LLMs have taken shape as dynamic regulation and interdisciplinary collaboration in the policy and ecological construction of management in chronic ocular disease (Huang X. Q. et al., 2024; Li J. J. et al., 2024), their clinical application is still constrained by high-risk regulatory loopholes, data silos at the grassroots level, and the chronic ocular diseases management relies on the *status quo* such as single-visit treatment. In the future, it is necessary to build a paradigm of “full-cycle management” based on LLMs through the dual-track strategy of “accurate regulation + open collaboration”, enforcing algorithmic traceability, fine-tuning risk and responsibility stratification, and expediting the formulation of specialized laws and regulations for LLMs in chronic ocular disease management, clarifying legal responsibilities and rights in various scenarios. Build a global ethical framework together and establish a standardized ethical review criterion, ensuring that the application of LLMs complies with ethical norms. Integrate home devices, electronic medical records and genetic data to build personalized prediction models (Guo et al., 2024), develop digital therapies with adaptive interventions, and form a closed loop of “monitoring-warning-intervention”, and ultimately upgrading LLMs into a smart core of total domain empowerment, which can actively intercept disease deterioration in chronic ocular diseases such as diabetic retinopathy through continuous biomarker tracking and behavioral interventions, and build a new eye health ecosystem that is safe, fair, and patient-centered.

## 6 Conclusion

A panoramic overview of LLMs applied research in chronic ocular diseases through a scoping review reveals the multidimensional potential and ecological challenges of this technology. In terms of disease screening and diagnosis, LLMs have demonstrated dynamic learning capabilities that can break through the traditional single-modality limitations and significantly improve accuracy; while in the dimension of accessibility of healthcare resources, the personalized health guides generated by LLMs are reshaping the path of health management for low-literacy groups. It is noteworthy that the scoping review also exposes challenges in the implementation of the technology: on the one hand, the generalization ability of complex clinical scenarios is limited by the lack of specialization of labeled data, which leads to the model easily falling into the predicament of “high accuracy in the lab - low robustness in the clinic”; on the other hand, there is no global consensus on the ethical risks, from the dynamic desensitization of patient privacy to the interpretability of algorithmic decision-making, the existing studies are mostly theoretical discussions, and there is a lack of practical frameworks that can be transferred to ophthalmology. In the future, it is necessary to build a “technology-clinical-policy” collaborative innovation system: optimize the efficient use of multicenter ophthalmic data through federated learning, develop more LLMs suitable for chronic ocular diseases management scenarios, and truly release their universal value in chronic ocular diseases applications, so as to promote the global eye health equity from the vision to the ground.
