# Supplementary material for: Large language models in the management of chronic ocular diseases: a scoping review
Source: Front Cell Dev Biol. 2025 Jun 18;13:1608988. doi: 10.3389/fcell.2025.1608988 (PMC12213593; doi:10.3389/fcell.2025.1608988)
Supplement: Supplementary file 1 [file Table1.docx]

**Supplementary Table 1** Types, applications and performance of LLMs in chronic ocular diseases

| **Disease** | **Model** | **Objective** | **Performance** |
| --- | --- | --- | --- |
| **Uveitis** | **ChatGPT-4** | **Answer 43 questions of multimodal imaging (Mihalache et al., 2024)** | **Accuracy (67%)** |
|  |  | **Answer 27 clinical questions from reputable sources (Zhao et al., 2024a)** | **Accuracy (88.9%), FKGL (15.01) Comprehensiveness (2.83)** |
|  |  | **Diagnose 6 clinical cases of different degrees (Rojas-Carabali et al., 2024b)** | **Accuracy (66%)** |
|  |  | **Diagnose 25 standardized cases based on SUN (Rojas-Carabali et al., 2024a)** | **Initial Diagnosis accuracy (60%) After Adding Top 2 Differentials (72%)** |
|  | **ChatGPT-3.5** | **Answer 27 clinical questions from reputable sources (Zhao et al., 2024a)** | **Accuracy (66.7%), FKGL (16.11) Comprehensiveness(N/A)** |
|  |  | **Answer 32 questions formulated by specialists (Marshall et al., 2024)** | **Median accuracy score (4/6) Median completeness score (2/3)** |
|  |  | **Diagnose 6 clinical cases of different degrees (Rojas-Carabali et al., 2024b)** | **Accuracy (66%)** |
|  |  | **Diagnose 25 standardized cases based on SUN (Rojas-Carabali et al., 2024a)** | **Initial Diagnosis accuracy (60%) After Adding Top 2 Differentials (72%)** |
|  | **Google Gemini** | **Answer 27 clinical questions from reputable sources (Zhao et al., 2024a)** | **Accuracy (40.7%), FKGL (11.53) Comprehensiveness(N/A)** |
|  | **Claude3** | **Answer 27 clinical questions from reputable sources (Zhao et al., 2024a)** | **Accuracy (96.3%), FKGL (15.35) Comprehensiveness (2.67)** |
|  | **Glass 1.0** | **Diagnose 6 clinical cases of different degrees (Rojas-Carabali et al., 2024b)** | **Accuracy (33%)** |
| **Myopia** | **ChatGPT-3.5** | **Decision on 100 pediatric refractive records (Kang et al., 2025)** | **Accuracy (85%), GQS (4.14)** |
|  |  | **Answer 20 questions from children in Chinese (Chang et al., 2024)** | **Cohen’s kappa (0.820), CRIE (7.29-12.09)** |
|  |  | **Response 31 nursing related problems in 6 areas (Lim et al., 2023)** | **61.3% "good" accuracy** |
|  | **ChatGPT-4.0** | **Response 19 Public health issues  (Wang et al., 2024b)** | **Comprehensiveness (4.566/5) Accuracy (4.487/5), Relevance (4.513/5)** |
|  |  | **Response 31 nursing related problems in 6 areas (Lim et al., 2023)** | **80.6% "good" accuracy** |
|  | **ChatGPT-4o** | **Decision on 100 pediatric refractive records (Kang et al., 2025)** | **Accuracy (90%), GQS (4.4)** |
|  | **WenxinYiyan** | **Decision on 100 pediatric refractive records (Kang et al., 2025)** | **Accuracy (80%), GQS (4.09)** |
|  | **Google Bard** | **Response 19 Public health issues  (Wang et al., 2024b)** | **Comprehensiveness (3.750/5) Accuracy (4.224/5), Relevance (4.342/5)** |
|  |  | **Response 31 nursing related problems in 6 areas (Lim et al., 2023)** | **54.8% "good" accuracy** |
|  | **Claude 2** | **Response 19 Public health issues  (Wang et al., 2024b)** | **Comprehensiveness (3.750/5) Accuracy (4.289/5), Relevance (4.368/5)** |
| **Amblyopia** | **ChatGPT-4** | **Response 27 questions in 2 times (Nikdel et al., 2024)** | **Acceptable responses: 81.5% Unacceptable responses:5.6%** |
| **Childhood Myopia** | **ChatGPT-4** | **Response 27 questions in 2 times (Nikdel et al., 2024)** | **Acceptable responses: 87.5% Unacceptable responses:5.4%** |
| **DED** | **ChatGPT-4** | **Creation based on 20 educational materials (Dihan et al., 2024c)** | **SMOG (4.5), DISCERN (4) FKGL (3.7), Likert (1)** |
|  |  | **Response 46 real patient interactions (Shi et al., 2024)** | **Patient Readability (4.61/5) Ophthalmologist Readability (2.67/5)** |
|  | **ChatGPT-3.5** | **Creation based on 20 educational materials (Dihan et al., 2024c)** | **SMOG (9.0), DISCERN (4) FKGL (9.5), Likert (1)** |
|  | **Gemini Advanced** | **Creation based on 20 educational materials (Dihan et al., 2024c)** | **SMOG (4.5), DISCERN (4) FKGL (3.7), Likert (1)** |
|  | **Baichuan 2** | **Response 46 real patient interactions (Shi et al., 2024)** | **Patient Readability (3.91/5) Ophthalmologist Readability (4.33/5)** |
| **Keratoconus** | **ChatGPT-4** | **Response 25 questions from Google searches (Demir, 2025)** | **DISCERN (65), FKCL (11.5) Likert scale mean score (4.72), FRE (32.5)** |
|  |  | **Response 20 questions from Google searches (Reyhan et al., 2024)** | **DISCERN (43.10), GQS (3.06) FRE (28.85), FKGL (14.64)** |
|  |  | **Response questions from 50 real patients (Kayabasi et al., 2024)** | **74% "strongly agreed" responses DISCERN (65), EQIP (67.5)** |
|  | **Google Gemini** | **Response 25 questions from Google searches (Demir, 2025)** | **DISCERN (58), FKCL (8.7) Likert scale mean score (4.40), FRE (40.8)** |
|  |  | **Response 20 questions from Google searches (Reyhan et al., 2024)** | **DISCERN (46.08), GQS (3.31) FRE (34.70), FKGL (12.46)** |
|  |  | **Response questions from 50 real patients (Kayabasi et al., 2024)** | **42% "strongly agreed" responses DISCERN (57), EQIP (67.5)** |
|  | **Copilot** | **Response 25 questions from Google searches (Demir, 2025)** | **DISCERN (55), FKCL (9.6) Likert scale mean score (4.24), FRE (34.6)** |
|  |  | **Response 20 questions from AAO (Özcan et al., 2025)** | **SOLO (3.75), PEMAT-U (66%) FRE (44.40), (PEMAT-A (40%)** |
|  |  | **Response 20 questions from Google searches (Reyhan et al., 2024)** | **DISCERN (46.95), GQS (3.24) FRE (29.60), FKGL (12.04)** |
|  |  | **Response questions from 50 real patients (Kayabasi et al., 2024)** | **34% "strongly agreed" responses DISCERN (50), EQIP (70)** |
|  | **ChatGPT-3.5** | **Response 20 questions from AAO (Özcan et al., 2025)** | **SOLO (3.5), PEMAT-U (66%) FRE (39.77), PEMAT-A (40%)** |
|  |  | **Response 20 questions from Google searches (Reyhan et al., 2024)** | **DISCERN (42.91), GQS (3.02) FRE (21.43), FKGL (15.41)** |
|  | **Bard** | **Response 20 questions from AAO (Özcan et al., 2025)** | **SOLO (4), PEMAT-U (83%) FRE (34.78), PEMAT-A (40%)** |
|  | **Chatsonic** | **Response 20 questions from Google searches (Reyhan et al., 2024)** | **DISCERN (43.97), GQS (3.10) FRE (24.40), FKGL (13.38)** |
|  | **Perplexity** | **Response 20 questions from Google searches (Reyhan et al., 2024)** | **DISCERN (45.96), GQS (3.01) FRE (22.30), FKGL (15.50)** |
| **Glaucoma** | **BERT** | **Predict need for surgery (EHRs,4512) (Hu and Wang, 2022)** | **AUROC=73.4%, F1=45.0%>29.9%(Ophthalmologist)** |
|  |  | **Analysis of patient needs from 10,892 Chinese posts (Fu et al., 2023)** | **Accuracy (0.891), F1-score (0.891),**  **AUC (0.931)** |
|  |  | **Encode 56 surgical records automatically (Lee et al., 2023)** | **Accuracy (88%)** |
|  | **RoBERTa** | **Predict need for surgery (EHRs,4512) (Hu and Wang, 2022)** | **AUROC=72.4%, F1=44.7%>29.9%(Ophthalmologist)** |
|  | **DistilBERT** | **Predict need for surgery (EHRs,4512) (Hu and Wang, 2022)** | **AUROC=70.2%, F1=42.5%>29.9%(Ophthalmologist)** |
|  | **BioBERT** | **Predict need for surgery (EHRs,4512) (Hu and Wang, 2022)** | **AUROC=70.1%, F1=41.7%>29.9%(Ophthalmologist)** |
|  | **Bard** | **Response 32 diagnostic and therapeutic questions (Sensoy and Citirik, 2024)** | **Accuracy (78.1%)** |
|  |  | **Rewrite 20 educational child education materials (Dihan et al., 2024b)** | **Rewrites: PEMAT (83.3%),**  **SMOG (7.4), FKGL (7.4)** |
|  | **ChatGPT-4** | **Response 33 key questions regarding gaps (Wu et al., 2024)** | **Accuracy: 1^st^ (66.7%), 2^nd^ (61%) Consistency: 48% differdence** |
|  |  | **Select surgical methods in 60 surgeries (Carlà et al., 2024)** | **Coherence with experts (58%)** |
|  |  | **Simplify 71 reading texts from journals and society  (Spina et al., 2025)** | **FKGL (⬇30%), FRE (⬆66%),**  **5th grade reading level** |
|  |  | **Diagnose 6 clinical cases in 2 profiles (Ming et al., 2024)** | **Registration accuracy: 60%>20%(Residents) Diagnostic accuracy: 100%=100%(Residents)** |
|  |  | **Response 34 questions in unsponsored website (Dogan and Yilmaz, 2025)** | **Appropriateness (88.2%),**  **FRE (28.8, 4.5% of U.S. adults)** |
|  |  | **Response 53 questions from websites and hospitals (Kerci and Sahan, 2024)** | **Correct (88.7%), Insufficient (7.5%), Misleading (3.8%)** |
|  |  | **Predict conversion of 1504 hypermetropia patients (Huang et al., 2024b)** | **Accuracy (75%), AUC (0.67),  Sensitivity (56%), Specificity (78%)** |
|  |  | **Compared with fellowship-trained expert diagnosis (Huang et al., 2024a)** | **Accuracy rank: 506.2>403.4 (Specialists) Completeness rank: 528.3>398.7 (Specialists)** |
|  |  | **Rewrite 20 educational child education materials (Dihan et al., 2024b)** | **Rewrites: PEMAT (83.3%),**  **SMOG (4.8), FKGL (3.7)** |
|  |  | **Answer 18 questions of multimodal imaging (Mihalache et al., 2024)** | **Accuracy (61%)** |
|  | **ChatGPT-4o** | **Diagnose 26 cases both primary and secondary types (Zhang et al., 2024)** | **Primary: Accuracy (5.5/10),**  **Completeness (3.077/10) Differential: Accuracy (4.096/10), Completeness (7.577/10)** |
|  | **ChatGPT-3.5** | **Diagnose 11 cases based on specific clinical scene (Delsoz et al., 2023)** | **Accuracy: 72.7%>54.5% (senior ophthalmology resident** |
|  |  | **Diagnose 6 clinical cases in 2 profiles (Ming et al., 2024)** | **Registration accuracy: 20%=20%(Residents) Diagnostic accuracy: 100%=100%(Residents)** |
|  |  | **Response 24 clinical questions in 4 categories (Tan et al., 2024)** | **Qualified (70.8%),**  **Need improvement (29.2%)** |
|  |  | **Predict conversion of 1504 hypermetropia patients (Huang et al., 2024b)** | **Accuracy (61%), AUC (0.62),  Sensitivity (64%), Specificity (59%)** |
|  |  | **Response 32 diagnostic and therapeutic questions (Sensoy and Citirik, 2024)** | **Accuracy (56.3%)** |
|  |  | **Rewrite 20 educational child education materials (Dihan et al., 2024b)** | **Rewrites: PEMAT (83.3%),**  **SMOG (7.1), FKGL (7.0)** |
|  | **Text-davinci-002** | **Generate health information about surgery (Kianian et al., 2024)** | **Readability (9.4 grade level)** |
|  | **Google Gemini** | **Select surgical methods in 60 surgeries (Carlà et al., 2024)** | **Coherence with experts (32%)** |
|  | **Bing Chat** | **Response 34 questions in unsponsored website (Dogan and Yilmaz, 2025)** | **Appropriateness (88.2%),**  **FRE (43.4, 33% of U.S. adults)** |
|  |  | **Response 32 diagnostic and therapeutic questions (Sensoy and Citirik, 2024)** | **Accuracy (59.4%)** |
|  | **Bi-LSTM** | **Analysis of patient needs from 10,892 Chinese posts (Fu et al., 2023)** | **Accuracy (0.82), F1-score (0.821),**  **AUC (0.890)** |
|  | **VLLM** | **Categorizing retinal fundus images in five datasets (Wang et al., 2024a)** | **REFUGE: Accuracy (0.9875),**  **Sensitivity (0.9) ORIGA: Accuracy (0.8308),**  **Sensitivity (0.7647) G1020: Accuracy (0.9853),**  **Sensitivity (0.9831) AI-HUB: Accuracy (0.9702),**  **Sensitivity (0.9722) Private: Accuracy (0.9322),**  **Sensitivity (0.9310)** |
| **Ocular Surface Diseases** | **ChatGPT-4** | **Answer 100 questions by an ophthalmology professor (Ling et al., 2025)** | **Average score:66>61.5(Resident)** |
|  | **ChatGPT-3.5** | **Answer 100 questions by an ophthalmology professor (Ling et al., 2025)** | **Average score:50<61.5(Resident)** |
|  | **Claude 2** | **Answer 100 questions by an ophthalmology professor (Ling et al., 2025)** | **Average score:46<61.5(Resident)** |
|  | **PaLM2** | **Answer 100 questions by an ophthalmology professor (Ling et al., 2025)** | **Average score:49<61.5(Resident)** |
|  | **SenseNova** | **Answer 100 questions by an ophthalmology professor (Ling et al., 2025)** | **Average score:48<61.5(Resident)** |
| **Corneal Eye Diseases** | **ChatGPT-4** | **Diagnose tentativly 20 cases (Delsoz et al., 2024)** | **Accuracy: 85%<90% (Human experts)** |
|  | **ChatGPT-3.5** | **Diagnose tentativly 20 cases (Delsoz et al., 2024)** | **Accuracy: 60%<90% (Human experts)** |
| **Age-relatedmacular Degeneration** | **ChatGPT-4** | **Response 15 questions in full-process (Cheong et al., 2024)** | **%"Good" Responses (73.3%)** |
|  |  | **Response 133 questions in 6 criterias (Muntean et al., 2024)** | **C₁(88.72%) C₂(97.74%) C₃(98.5%) C₄(100%) C₅(99.25%) C₆(91.73%)** |
|  | **ChatGPT-3.5** | **Response 15 questions in full-process (Cheong et al., 2024)** | **%"Good" Responses (80.0%)** |
|  | **Google Bard** | **Response 15 questions in full-process (Cheong et al., 2024)** | **%"Good" Responses (40.0%)** |
|  | **OcularBERT** | **Response 15 questions in full-process (Cheong et al., 2024)** | **%"Good" Responses (13.3%)** |
|  | **PaLM2** | **Response 133 questions in 6 criterias (Muntean et al., 2024)** | **C₁(60.90%) C₂(85.71%) C₃(86.47%) C₄(93.23%) C₅(84.96%) C₆(69.92%)** |
| **Ocular oncology** | **ChatGPT-4** | **Answer 21 questions of multimodal imaging (Mihalache et al., 2024)** | **Accuracy (72%)** |
| **Retinal Diseases** | **ChatGPT-4** | **Diagnose 6 clinical cases in 2 profiles (Ming et al., 2024)** | **Registration accuracy: 95%>91%(Residents) Diagnostic accuracy: 57%<60%(Residents)** |
|  |  | **Compared with fellowship-trained expert diagnosis (Huang et al., 2024a)** | **Accuracy rank: 235.3>216.1 (Specialists) Completeness rank: 258.3>208.7 (Specialists)** |
|  |  | **Answer 209 questions of multimodal imaging (Mihalache et al., 2024)** | **Accuracy (77%)** |
|  | **ChatGPT-3.5** | **Diagnose 6 clinical cases in 2 profiles (Ming et al., 2024)** | **Registration accuracy: 86%<91%(Residents) Diagnostic accuracy: 37%<60%(Residents)** |
|  | **BERT** | **Encode 298 surgical records automatically (Lee et al., 2023)** | **Accuracy (88%)** |
| **Cataract** | **ChatGPT-3.5** | **Response 46 questions related to care (Su et al., 2025)** | **Accuracy (95.65%),**  **Comprehensiveness (13/15)** |
|  |  | **Response 20 questions from Google searches (Cohen et al., 2024)** | **Accuracy (94%) Readability (college level))** |
|  |  | **Resident Training Development Exam (Balci et al., 2024)** | **Accuracy(53.62%）** |
|  |  | **Generate information from large datasets (Yilmaz and Dogan, 2025)** | **SOLO (3.1), FK (34.38)** |
|  | **Google Bard** | **Generate information from large datasets (Yilmaz and Dogan, 2025)** | **SOLO (2.9), FK (55.5)** |
|  | **ChatGPT-4o** | **Response 46 questions related to care (Su et al., 2025)** | **Accuracy (100%),**  **Comprehensiveness (13.22/15)** |
|  | **Google Bard** | **Response 46 questions related to care (Su et al., 2025)** | **Accuracy (97.83%),**  **Comprehensiveness (13/15)** |
|  | **Bing AI** | **Generate information from large datasets (Yilmaz and Dogan, 2025)** | **SOLO (2.65), FK (41.77)** |
|  | **Bing Chat** | **Response 46 questions related to care (Su et al., 2025)** | **Accuracy (89.13%),**  **Comprehensiveness (10.9/15)** |
|  | **slit lamp-GPT** | **Generate reports from 25,051 images (Zhao et al., 2024b)** | **BLEU (0.67, 0.66, 0.65, 0.6) CIDE (3.24), SPICE (0.37)** |
|  | **Llama 2** | **Response 46 questions related to care (Su et al., 2025)** | **Accuracy (69.57%),**  **Comprehensiveness (12.4/15)** |
|  | **Wenxin Yiyan** | **Response 46 questions related to care (Su et al., 2025)** | **Accuracy (60.87%),**  **Comprehensiveness (9.13/15)** |
|  | **BERT** | **Encode 374 surgical records automatically (Lee et al., 2023)** | **Accuracy (88%)** |
| **Macular degeneration** | **BERT** | **Encode 49 surgical records automatically (Lee et al., 2023)** | **Accuracy (88%)** |
| **Diabetic Retinopathy** | **ChatGPT-4** | **Response 15 questions in full-process (Cheong et al., 2024)** | **%"Good" Responses (93.3%)** |
|  |  | **Response 20 expert questions (Subramanian et al., 2024)** | **Appropriateness score: 4.84/5 Completeness score: 4.38/5** |
|  | **ChatGPT-3.5** | **Response 15 questions in full-process (Cheong et al., 2024)** | **%"Good" Responses (93.3%)** |
|  | **ChatGPT-3.5-turbo** | **Answer in ten open-ended clinical scenarios (Maywood et al., 2024)** | **Accuracy (100%), obsolescence** |
|  | **Google Bard** | **Response 15 questions in full-process (Cheong et al., 2024)** | **%"Good" Responses (60.0%)** |
|  | **OcularBERT** | **Response 15 questions in full-process (Cheong et al., 2024)** | **%"Good" Responses (6.7%)** |
|  | **DR-GPT** | **Categorize 31292 patients for severity (Jaskari et al., 2024)** | **QWK= 0.975, accuracy (98.7%)** |
|  | **BERT** | **Extract clinical concept from 536 image reports (Yu et al., 2022)** | **Strict F1=0.8578, Lenient F1=0.8881** |
|  |  | **Encode 149 surgical records automatically (Lee et al., 2023)** | **Accuracy (88%)** |
|  | **RoBERTa** | **Extract clinical concept from 536 image reports (Lee et al., 2023)** | **Strict F1=0.8578, Lenient F1=0.8861** |
| **Paediatric Cataract** | **ChatGPT-3.5** | **Rewrite 20 education materials (Dihan et al., 2024a)** | **SMOG (7.31), FKGL (6.86)** |
|  | **ChatGPT-4** | **Rewrite 20 education materials (Dihan et al., 2024a)** | **SMOG (5.14), FKGL (3.76)** |
|  | **Google Bard** | **Rewrite 20 education materials (Dihan et al., 2024a)** | **SMOG (7.67), FKGL (7.26)** |
| **Lens Diseases** | **ChatGPT-4** | **Diagnose 4 clinical cases in 2 profiles (Ming et al., 2024)** | **Registration accuracy: 50%>25%(Residents) Diagnostic accuracy: 75%>50%(Residents)** |
|  | **ChatGPT-3.5** | **Diagnose 4 clinical cases in 2 profiles (Ming et al., 2024)** | **Registration accuracy: 25%=25%(Residents) Diagnostic accuracy: 25%<50%(Residents)** |
| **Vitreoretinal Disease** | **ChatGPT-4V** | **Subtype distinguish in 256 eyes (Ghalibafan et al., 2024)** | **Open-ended questions (13.7%) Multiple-choice questions (31.3%)** |
| **Neovascular Age-related macular Degeneration** | **ChatGPT-3.5-turbo** | **Answer in ten open-ended clinical scenarios (Maywood et al., 2024)** | **Accuracy (86.7%), potential hazards** |
| **Retinal vascular disease** | **ChatGPT-3.5-turbo** | **Answer in ten open-ended clinical scenarios (Maywood et al., 2024)** | **Accuracy (76.7%), Identify hazards** |
